# The interface of COVID-19, diabetes, and depression

**DOI:** 10.1007/s44192-022-00007-0

**Published:** 2022-03-01

**Authors:** Charlotte Steenblock, Peter E. H. Schwarz, Nikolaos Perakakis, Naime Brajshori, Petrit Beqiri, Stefan R. Bornstein

**Affiliations:** 1 Department of Internal Medicine III, University Hospital Carl Gustav Carus, Technische Universität Dresden, Fetscherstrasse 74, 01307 Dresden, Germany; 2 Paul Langerhans Institute Dresden (PLID), Helmholtz Center Munich, University Hospital and Faculty of Medicine Carl Gustav Carus, TU Dresden, Dresden, Germany; 3 German Center for Diabetes Research (DZD E.V.), Neuherberg, Germany; 4 Research Unit, Heimerer College, Prishtina, Republic of Kosovo; 5 Department of Endocrinology, Diabetology and Clinical Nutrition, University Hospital Zurich, Zurich, Switzerland; 6 Department of Diabetes, School of Life Course Science and Medicine, Kings College London, London, UK

## Abstract

Comorbid diabetes with depression is a challenging and often under-recognized clinical problem. During the current COVID-19 pandemic, a communicable disease is thriving on the increasing incidences of these non-communicable diseases. These three different health problems are bidirectionally connected forming a vicious cycle. Firstly, depressed individuals show a higher risk of developing diabetes and patients with diabetes have a higher risk of developing symptoms of depression. Secondly, patients with diabetes have a higher risk of developing severe COVID-19 as well as of experiencing breakthrough infections. Thirdly, in both patients with type 2 diabetes and in COVID-19 survivors the prevalence of depression seems to be increased. Fourthly, lockdown and quarantine measurements during the COVID-19 pandemic has led to an increase in depression. Therefore, it is of importance to increase the awareness of this interface between depression, diabetes and COVID-19. Finally, as symptoms of post-COVID, diabetes and depression may be overlapping, there is a need for educating skilled personnel in the management of these comorbidities.

## Introduction

Infectious and metabolic diseases, such as diabetes, are major health problems worldwide [1]. Likewise, the high prevalence of depression is considered a global health burden [2]. Moreover, comorbid diabetes and depression is a challenging and often under-recognized clinical problem [3, 4].

In the last decades, medical advances, access to better health care (e.g. vaccination) and improved sanitation have reduced the overall mortality and morbidity due to infectious diseases, in particular for lower respiratory tract infections and diarrheal disease [5]. Nevertheless, mortality and morbidity as a result of neglected tropical diseases, HIV infection, tuberculosis and malaria remain high in low-income countries [5]. Furthermore, the number of deaths due to new and re-emerging infections in addition to seasonal and endemic infections have remained relatively constant in the twenty-first century [5]. In addition, in the last decades, the spreading-speed of infectious diseases has escalated unprecedently due to technological, demographic and climatic changes: people travel more, more people live in urban than in rural areas and the population numbers are continuously increasing [5]. A phenomenon that unfortunately has been further observed during the current COVID-19 pandemic.

Harmful interactions between infectious and metabolic diseases that involve the immune system and multiple other organ systems (e.g. pancreas, adrenal glands, adipose tissue, liver, and gut) are present [6]. For example, development of acute diabetes after viral infections, such as Mumps, Coxsackie B3 and B4, Rubella, and Influenza B, has been reported [7]. In this instance, insulin-producing beta-cells are damaged due to the direct infection without autoimmunity [7, 8]. An alternative mechanism might be due to chronic, repeated viral exposure. This mechanism includes molecular mimicry, where viral epitope sequences bear resemblance to beta-cell antigens e.g. viral peptides and insulin. Thereby, they can potentially trigger a cross-reactive autoimmune response [9, 10]. These interactions are bidirectional and have a major impact on metabolic or infectious disease development, severity, duration, response to treatment and consequently on quality of life and mortality. The overwhelming dimension of this problem has become evident in the current COVID-19 pandemic, in which we have witnessed a communicable viral pandemic with the existing non-communicable pandemic of metabolic diseases affecting a significant percentage of the world population [11].

On top of this, the number of people diagnosed with major depression is steadily increasing. In 2019, it was found that globally depression caused 46.9 million Disability Adjusted Life Years (DALYs) [1]. Moreover, the majority of depression cases remain undiagnosed or untreated, a problem that is more severe in low- and middle-income countries [12].

**Fig. 1:**
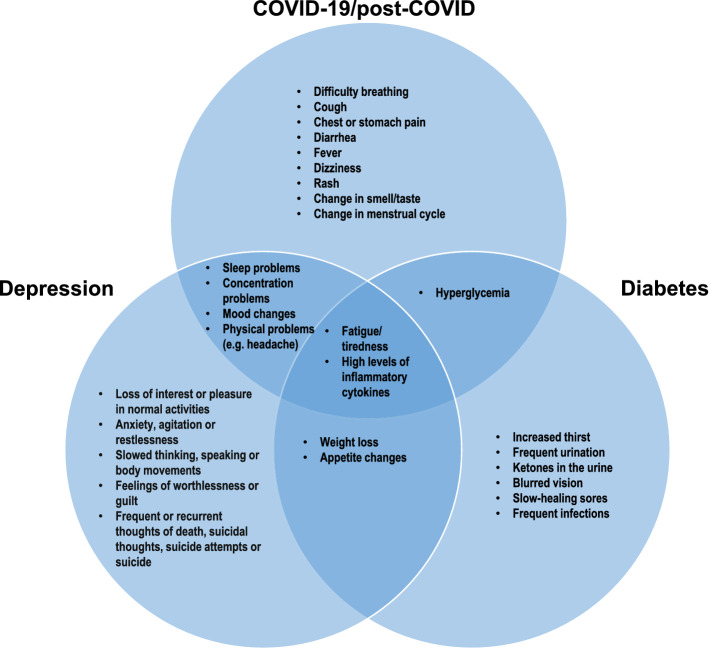
Interface of COVID-19, diabetes and depression. Symptoms of these three health problems are partly overlapping indicating the importance of educating skilled personnel to detect and diagnose these symptoms in order to employ the right treatment

A number of symptoms connected to post-COVID, diabetes and depression, such as fatigue and increased levels of cytokines, are similar (Fig. 1). In the current narrative review, we will discuss what is already known about the interface between COVID-19, diabetes and depression and discuss the challenges for health personal to identify the symptoms and to prevent long-term consequences. First, we describe the interaction between diabetes and depression, then we focus on patients with diabetes and the impact of COVID-19 on this group of patients. Afterwards, we discuss how COVID-19 can lead to depression and at the end, we highlight the interaction between diabetes, depression and COVID-19. In our conclusion, we discuss how important it is to be aware of this vicious cycle and to educate skilled personnel.

For preparation of the manuscript, we searched PubMed and Google Scholar for articles published up to January 20, 2022. Search items included “COVID-19”, “post-COVID”, “long COVID”, “SARS-CoV-2”, “diabetes”, “hyperglycaemia”, “hyperglycemia”, “insulin”, and “depression”. The reference list of original articles, narrative reviews, clinical guidelines, position statements, systematic reviews, and meta-analyses was screened for applicable publications. The final reference list was selected based on relevance to the topic of this Review with preference given to the most recent relevant publications.

## Diabetes and depression

In 2019, the global diabetes prevalence was estimated to be 9.3% (463 million people), rising to 10.2% (578 million) by 2030 and 10.9% (700 million) by 2045. The prevalence was higher in urban (10.8%) than in rural (7.2%) areas. Furthermore, the frequency was much higher in high-income (10.4%) than in low-income countries (4.0%) [13].

Depression comprises a number of emotional, cognitive, and behavioural or somatic symptoms (Fig. 1) [14]. Depression has a direct or indirect relation with a number of diseases including Alzheimer’s disease, stroke, epilepsy, diabetes, cardiovascular disease and cancer [15], and the prevalence of depression is significantly higher in individuals suffering from a chronic disease than in those without [14].

Diabetes is associated with an increased risk of depression [16–19]. At the same time, people with depression have a more than 30% higher risk of developing diabetes than people without depression [20, 21]. The underlying pathophysiological mechanisms behind this bidirectional relationship between diabetes and depression are not fully elucidated but inflammatory mechanisms and insulin resistance seem to play an important role [4]. Both diabetes and depression are associated with a chronic state of systemic low-grade inflammation, and a recent meta-analysis demonstrated that the blood concentrations of C-reactive protein and interleukin-6 (IL-6) were higher in people with type 2 diabetes and comorbid depression than in patients with type 2 diabetes but without depression [22]. On the other hand, the peripheral blood concentration of brain derived neurotrophic factor (BDNF) was lower among the patients with comorbid diabetes and depression [22].

Another shared manifestation of diabetes and depression is the dysregulation of the hypothalamic–pituitary–adrenal cortex (HPA) [23]. This pathway is important under stress and regulate immune function, glucose metabolism and sleep, which are indicators altered in both diseases (Fig. 1) [24]. Under stress, when the HPA axis is activated, corticotropin-releasing hormone (CRH) and arginine vasopressin (AVP) are secreted from the paraventricular nucleus of the hypothalamus. CRH and AVP stimulate the anterior pituitary to secrete adrenocorticotropic hormone (ACTH), and via the bloodstream, ACTH leads to the production of glucocorticoids and mineralocorticoids in the adrenal cortex. The cortisol secreted from the adrenal cortex will subsequently increase blood glucose levels by stimulating gluconeogenesis in the liver and by decreasing glucose uptake in muscle and white adipose tissue, thus antagonizing the effects of insulin on glucose homeostasis. This may further aggravate insulin resistance, increase hunger and lead to weight gain and hyperglycemia [25]. During acute stress, this response is crucial for survival, whereas chronic stress, as in depression, can have harmful effects. Eventually, long-term chronical activation of the HPA axis with elevated cortisol levels may lead to cortisol dysregulation and altered feedback control mechanisms. These changes have been associated with type 2 diabetes and depression [26, 27]. Metabolic stress, as often observed in depression, may also lead to activation of the HPA axis as we have recently observed in mice, where high-fat-diet leads to a hypertrophy of the adrenal gland and hyperactivation of the HPA axis [28].

The co-occurrence of diabetes and depression has not only direct but also indirect consequences on human health due to its impact on patient compliance and self-motivation. Specifically, depression substantially impairs the quality of life in patients with diabetes and more intensive support for diabetes management is required as self-management becomes more challenging [29]. Motivating and convincing patients with diabetes to change lifestyle and adhere to treatment is often already a demanding task. If these patients suffer additionally from depression, it becomes even more complicated.

Diabetic complications, insulin use and educational status have been identified as risk factors for co-morbid depression in patients with type 2 diabetes, whereas regular exercising, gender, marital status and current social status were demonstrated to be protective factors [30]. Furthermore, age is a common risk factor for both diabetes and depression [31]. Thereby women, people with a low educational level and people living in rural areas are at a higher risk, whereas being married and doing regular exercise protect from developing comorbid depression in patients with type 2 diabetes [17, 30]. Thus, with the increasing life expectations, diabetes and depression represent a serious burden on the medical care systems. Therefore, it is important to promote screening activities and introduce targeted and personalized treatment for depression in order to reduce the risk of poor short- and long-term outcomes of diabetes [17].

## COVID-19, post-COVID and diabetes

Diabetes and other metabolic diseases increase the risk of developing severe COVID-19 [11]. In patients with diabetes, impaired insulin signalling might lead to chronic subclinical low-grade inflammation via activation of AP-1 and NF-κB leading to a decrease in anti-inflammatory cytokines and to an increase in the pro-inflammatory cytokines TNF-α, IL-6 and IL-1β. These cytokines inhibit insulin signalling [32], thereby boosting insulin resistance [33]. In severe COVID-19, the inflammatory response to SARS-CoV-2 infection can further promote insulin resistance and endothelial dysfunction [11].

Patients with diabetes do not only have a higher risk of developing severe COVID-19, they are also more prone to serious long-term consequences [34, 35], as COVID-19 may lead to an aggravation of underlying metabolic diseases and to new-onset-diabetes [36]. New-onset hyperglycaemia, ketoacidosis, diabetes and severe metabolic complications of pre-existing diabetes were repeatedly observed in patients with COVID-19 [37–43]. Patients with abnormalities in their blood glucose levels associated to COVID-19 had increased markers of inflammation and organs’ injury and poorer clinical outcome compared to those with normoglycemia [44]. In the pancreas, a direct infection with SARS-CoV-2 of the islets has been shown in several studies [45–47], which, as we have demonstrated, might lead to islet damage and necroptosis [46]. Even in utero, we observed viral infiltration of several organs including the pancreas [48].

In relation to diabetes, long-term consequences of COVID-19 designated as “post-COVID”, “long COVID” syndromes [49] or post-acute sequelae of COVID-19 [50], have been observed in a number of studies. One study investigating COVID-19 patients 1 year after recovery and hospital discharge, showed that ~ 1% of these patients developed new-onset diabetes and in ~ 10% of the patients, a worsening in glycaemic control was documented [51]. One Italian study showed that glycaemic abnormalities could be detected at least 2 months after recovery from COVID-19 [41], whereas another study recently showed that the prevalence of dysglycaemia reverted to pre-admission frequencies in most recovered patients [44].

Currently, the number of breakthrough infections with SARS-CoV-2 despite full vaccination are increasing. These patients that are infected even though they are fully vaccinated mostly experience mild symptoms. However, the vaccinated patients that do develop severe symptoms of COVID-19 are often elderly and frequently they suffer of comorbidities such as hypertension, diabetes, congestive heart failure and chronic kidney disease [52] indicating the importance of a booster vaccination especially for these patient groups.

## COVID-19 and depression

The COVID-19 pandemic seriously affects mental health. During a pandemic, the number of people whose mental health is affected actually tends to be higher than the number of people affected by the infection [53]. Prolonged exposure to stressors, such as those experienced through social isolation, concern of being infected, and loss of relatives or friends, increases the risk of developing major depression, anxiety and post-traumatic stress disorders [24]. As mentioned above, this is due to the excessive or chronic activation of the HPA axis, which also leads to a disrupted feedback loop [54].

Several studies are currently emerging showing that social loneliness during COVID-19 lockdowns has had a negative influence on the development of mental diseases in all age groups. Across the world, childhood obesity increased during the pandemic. This was due to changes in the daily routine such as a reduction in physical activity and negative changes in the eating habits during lockdown. This also had a negative impact on psychological well-being [55–57]. In adolescents, COVID-19 has dramatically altered the number of social contacts by limiting in-person interactions. Thus, it was shown that particularly girls and individuals with a depressive disorder are at an increased risk of suffering from pandemic-associated psychological distress [58]. A similar pattern was observed with students (18–29 years) in Columbia after social isolation in the COVID-19 pandemic, where women exhibited a higher risk of developing depression symptoms than men [59]. Furthermore, childless people and individuals with low income were more likely to display symptoms of depression [59]. Another study among university students in Germany showed that during the pandemic (June 2020), a small increase in depression was observed, whereas anxiety and somatic complaints did not change significantly compared to before the pandemic (June–August 2019) [60]. A meta-analysis revealed that in adults there was a significant relationship between quarantine and mental health during the COVID-19 pandemic with the longer the quarantine time, the higher the anxiety, depression and stress levels [61]. Between countries, no differences were observed [61] further pointing out that this is truly a global problem. A study with individuals aged 70 and older showed that in this age group lockdown led to a significant worsening of perceived stress, well-being, depressive symptoms, mood disturbance and memory. On the other hand, significant improvements in self-reported physical health symptoms, social interaction, time spent engaging in physical activity and certain aspects of relationship quality were demonstrated [62]. Follow-up showed that well-being, depression and mood were still negatively affected post-lockdown [62].

Mental health problems that manifested during the pandemic were predominantly associated with current loneliness and pre-pandemic distress [60]. A recent study among health care providers in Kosovo showed that interventions to minimize COVID-19-related mental health consequences by transforming experienced trauma into something positive by improving the coping skills could be highly beneficial in pandemic response work [63]. Overall, these studies underline the importance of adequate mental health care options during quarantine and lockdown. Recent results suggest telehealth to be a feasible care alternative as no significant differences were observed between in-person and telehealth groups in depressive symptom reduction [64].

Growing data indicate that SARS-CoV-2 can directly infiltrate the central nervous system and peripheral nervous system causing multiple neurological diseases. Increased incidences of psychiatric disorders have been observed during and after an infection with SARS-CoV-2 [65]. Most of these patients displayed high levels of pro-inflammatory cytokines [66]. However, a recent systematic review indicates that long-term mental effects from direct COVID-19 infection are associated with no or mild symptoms [67]. Studies exhibiting long-term prevalence of anxiety, depression, PTSD, and sleep disturbances were comparable to the levels observed in the general population [67].

## Interface between COVID-19, diabetes and depression

**Fig. 2:**
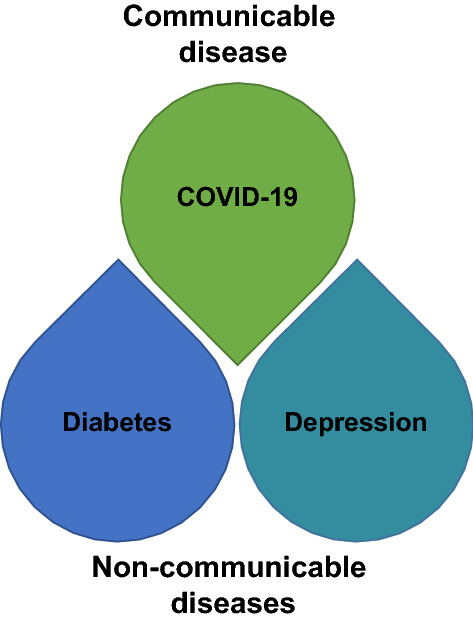
COVID-19, diabetes and depression form a vicious cycle. On one hand, diabetes increase the risk of severe COVID-19. On the other hand, COVID-19 may lead to new-onset diabetes or worsening of already existing metabolic disorders. On top of this, depressed individuals show a higher risk of developing diabetes and patients with diabetes have a higher risk of developing symptoms of depression. Furthermore, in patients with type 2 diabetes and in COVID-19 survivors the prevalence of depression is increased. Finally yet importantly, lockdown and quarantine measurements during the COVID-19 pandemic has led to an increase in depression

During the COVID-19 pandemic, we have observed that patients with diabetes have a significantly higher risk of contracting COVID-19 associated with an increased morbidity and mortality [11, 34]. The modulating factor for patient’s mortality seems to be insulin resistance especially in patients with obesity and type 2 diabetes [11]. Surviving COVID-19, a growing number of patients contract post-COVID-associated fatigue-syndrome [68]. The symptoms of fatigue-syndrome are very similar to depressive symptoms especially in patients with type 2 diabetes (Fig. 1). This vicious circle between diabetes, COVID-19, and depression might be associated with increased mortality (Fig. 2).

Health care avoidance during the COVID-19 pandemic has especially been observed in certain population groups. A population-based cross-sectional study in the Netherlands showed that one in five individuals avoided healthcare during lockdown in the COVID-19 pandemic, often for potentially urgent symptoms. Healthcare avoidance was strongly associated with female sex, fragile self-appreciated health, and high levels of depression and anxiety [69]. These results further emphasize the need for targeted public education urging these vulnerable patient groups to timely seek medical care for their symptoms to mitigate major health consequences.

A number of studies have recently focused on individuals with diabetes and the development of diabetes-related emotional distress and/or depression [70–74]. In a German study, it was shown that in patients with diabetes, the initial COVID-19 outbreak had a negative impact on the mental health status in these patients [73]. A similar conclusion was reached in a Brazilian study, where a high degree of psychological distress amongst patients with diabetes was reported [70]. The same working group showed an increase in suicidal thoughts in patients with diabetes 1 year after the COVID-1 outbreak [71]. A study from Saudi Arabia, showed no difference between the prevalence of depression and anxiety symptoms during the COVID-19 outbreak in people with or without diabetes [72]. However, certain subgroups of patients with diabetes showed a higher prevalence than people without diabetes. These subgroups included people who experienced canceling of their diabetes visit by the clinic, or patients that had no method of telecommunication with their health care providers. Furthermore, patients, which in addition to their diagnosis of diabetes, had HbA1C of ≥ 10%, were women, employees (in particular health care providers), students, unmarried individuals, and those with lower income were more likely to report depression and/or anxiety symptoms [72].

These results indicate the importance of developing strategies to mitigate the negative effects on mental health in especially patients with diabetes during the COVID-19 pandemic. One possibility would be to use telecommunication, which has been shown to have the potential to reduce diabetes-related emotional distress [75]. Thereby, the pandemic has presented people with diabetes and their healthcare providers with an opportunity to innovate and use digitalised care for home support [76].

After acute catastrophes such as a terror attack or a big fire, psychological assistance task forces for victims and their families are usually quickly organized [53]. However, during a pandemic, it is common for health professionals, scientists and managers first to focus on the pathogen and the biological risk in order to better understand the pathophysiological mechanisms involved and to propose measures for preventing, containing and treating the disease. In such situations, the psychological and psychiatric implications secondary to the phenomenon tend to be underestimated or even neglected. Thereby, an unnecessary burden of associated diseases might arise [77, 78].

In the light of the corona pandemic with a higher morbidity and mortality for those patients with diabetes and the growing presence of post-COVID and fatigue-syndrome, the early diagnosis of diabetes-associated depression and the effective management of fatigue-syndromes has become an urgent need. The presence of skilled personnel able to identify early signs of metabolic and/or psychological derangement may prove to be especially useful for preventing long-term complications and fatigue syndrome by facilitating the early initiation of multimodal treatment interventions.

For the future, this requires investment into education of medical personnel including nurses and the whole care team. Training nurses to identify early signs of high-risk medical conditions but also indicators of post-COVID symptoms such as fatigue may become an integral part of the medical care team.

The positive relationship between physical exercise, diabetes and mental health is well-established, but during the COVID-19 pandemic, with various restrictions, the space and facilities for physical exercise are limited. A recent review emphasizes how important it is to perform physical exercise anyway [79]. Especially, supervised physical exercises during COVID-19 were shown to be conducive to enhancing happiness and improving mental health. Furthermore, physical exercise reduced people’s anxiety, sadness and depression during the COVID-19 pandemic dependent on the intensity and frequency of physical exercise [79].

## Conclusion

Non-communicable diseases are a global challenge, accounting for 71% of all deaths worldwide [80]. The spread of COVID-19 and past huge disasters have affected the prevention and treatment of these diseases and require urgent action. Especially, the non-communicable diseases, depression and diabetes, are huge problems as these are directly or indirectly worsened either due to the actual infection or due to social limitations during lockdown or quarantine. A limitation of the current narrative review is that specific mechanisms explaining these correlations are not fully understood yet [4, 81, 82]. Still, symptoms of post-COVID, diabetes and depression are partly similar (Fig. 1) making it important with skilled personnel to detect and diagnose these symptoms in order to employ the right treatment.

## Data Availability

Not applicable.
